# Adrenal Gland Incidentaloma: A Rare Case of Extranodal B-cell Lymphoma

**DOI:** 10.7759/cureus.53231

**Published:** 2024-01-30

**Authors:** Inês Figueiredo, Francisco Guimarães, Cristina Duarte, Luísa Fontes

**Affiliations:** 1 Department of Internal Medicine, Hospital Companhia União Fabril (CUF) Descobertas, Lisboa, PRT

**Keywords:** r-chop therapy, rituximab, adrenalectomy, diffuse large b-cell lymphoma, adrenal gland incidentaloma

## Abstract

The prevalence of adrenal incidentalomas (i.e., incidental findings) has grown in recent years with the evolution of imaging methods. Adrenal masses can be benign or malignant. Malignant ones are less frequent, but the detection of primary adrenal neoplasms is even less frequent, especially in the case of a diffuse large B-cell lymphoma (DLBCL). This case concerns a 68-year-old man who presented to the emergency department due to fatigue and anorexia. Given his blood test results on admission, he underwent a computed tomography (CT) with angiography that identified a mass in the left adrenal gland with displacement of the ipsilateral kidney. Left tumorectomy, adrenalectomy, and nephrectomy were performed, and the mass corresponded to a nongerminal center-type DLBCL. This case highlights the importance of prompt diagnosis and surgical and pharmacologic treatment of DLBCL.

## Introduction

Adrenal masses are often incidental findings (incidentalomas) due to radiology exams driven by other medical conditions. In this sense, its incidence has increased in recent years due to widespread access to imaging modalities such as computed tomography (CT) and magnetic resonance imaging (MRI). The true prevalence of adrenal masses is around 6% [1]. Most incidentalomas represent benign masses, with a risk of malignancy of approximately 0.2% [2]. Among the malignant causes of incidentalomas, primary adrenal lymphomas are mostly described by case reports owing to their exceptional rarity, especially without the involvement of other lymph nodes [3]. In 2020, fewer than 250 cases of primary adrenal lymphoma were reported, and the diagnosis requires lymphomatous involvement of adrenal glands with no previous history of lymphoma [4]. It is also more common in male patients with an estimated ratio of 6:1 [5].

Although the adrenal gland has no lymphoid tissue, primary adrenal lymphoma is identified in less than 1% of non-Hodgkin lymphoma cases [6]. As a type of extranodal lymphoma, extranodal adrenal lymphoma is even rarer and constitutes less than 1% of extranodal lymphoma case reports [6]. Primary adrenal lymphoma is usually bilateral, and diffuse large B-cell lymphoma (DLBCL) is the most common subtype, accounting for approximately 70% of cases [6]. This lymphoma appears mainly in male patients, with an average age at diagnosis of 70 years old [6]. Symptoms are usually vague, with diffuse abdominal pain, weight loss, fever, and, in advanced cases, adrenal insufficiency. However, these symptoms usually appear later in the course of the disease, when there is an invasion of more than 90% of the adrenal gland [6]. The prognosis was very poor until the widespread introduction of new therapeutic schemes, with a one-year survival rate of 17.5% before the introduction of rituximab [6]. We present the case of a 68-year-old man diagnosed with a DLBCL of the adrenal gland that required excisional surgery for a definitive diagnosis.

## Case presentation

A 68-year-old male patient with a history of type 2 diabetes and prostate adenocarcinoma presented to the Emergency Department due to fatigue and anorexia that had worsened in the last three weeks. On admission, he was alert and oriented, was eupneic on room air, and had pale membranes. His blood pressure profile was controlled, he was apyretic, and he had no other remarkable findings on physical examination.

His laboratory evaluation revealed his hemoglobin was 11.6 g/dL, his white blood cell count was low, with 2100 leukocytes per microliter and 180,000 platelets per microliter, and his D-dimers increased by more than three times the upper limit of the reference range (2232 mcg/L, considering reference value < age x 10 mcg/L). His urea, creatinine, ionogram, and liver enzymes were within reference limits, but his C-reactive protein was 1.83 mg/dL (reference <1 mg/dL). There was no evidence of adrenal insufficiency.

Regarding his elevated D-dimers, a CT scan with angiography of the thorax excluded pulmonary thromboembolism but revealed a mass measuring 13.9 x 10.4 x13 cm in the left adrenal gland. The mass was heterogeneous and caused a caudal displacement of the ipsilateral kidney. There was also a small amount of free fluid in the left subphrenic space, suggesting the possibility of recent hemorrhage. The patient was admitted to the Internal Medicine Department for symptomatic control and etiological investigation.

**Figure 1:**
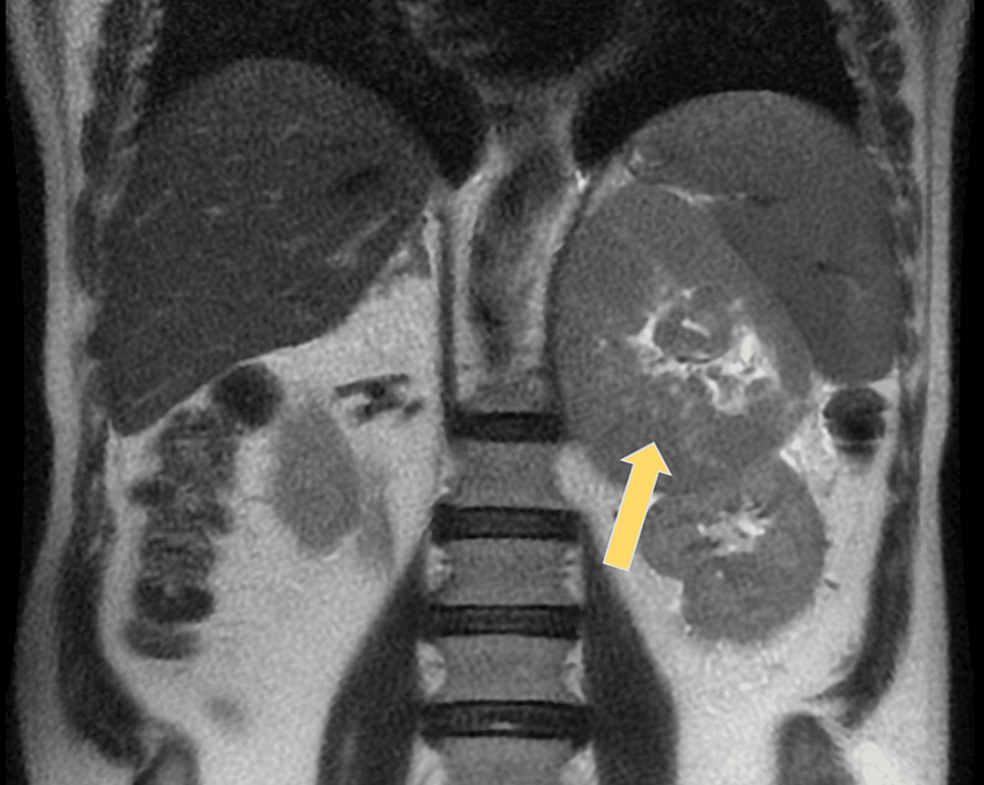
Abdominal magnetic resonance imaging The yellow arrow indicates the adrenal mass.

During his hospitalization, an abdominal and pelvic MRI corroborated the CT findings (Figure 1), revealing a solid and heterogeneous neoformative lesion in the left adrenal gland measuring 14 x 10 cm. No ganglia with increased dimensions were observed in the abdominal and pelvic ganglionic chains.

Considering the most likely diagnosis of an adrenal tumor, the patient underwent some endocrinological tests to identify whether it was a functioning or nonfunctioning tumor and determine its primary origin. His plasmatic cortisol, adrenocorticotrophic hormone, serum aldosterone, serum renin, plasma metanephrine, and catecholamine results were within reference ranges. Also, his 24-hour urine, vanilmandelic acid, urinary metanephrines, and urinary catecholamines were within the reference ranges. Protein electrophoresis and the summary urine test did not show alterations, and his urinary cytology test was negative for neoplastic cells. To exclude possible dissemination and define a therapeutic and prognostic strategy, we performed a cervical and thyroid ultrasound which revealed a normal thyroid without pathological ganglia. A myelogram and bone biopsy were performed to investigate possible medular involvement, and these also had no remarkable findings.

As it was a nonfunctioning adrenal tumor, the patient underwent a left tumorectomy, adrenalectomy, and nephrectomy. These procedures went without complications, and the surgical sample was sent to the Pathological Anatomy Department, which identified a DLBCL of the adrenal gland of the nongerminal center type with CD-20, Bcl-2, MUM-1, and Bcl-6 expressions.

After this diagnosis, the patient started a chemotherapy regimen with rituximab, cyclophosphamide, doxorubicin, vincristine, and prednisone (R-CHOP). His condition responded well, and 12 months after diagnosis, the patient continues to be in remission and undergoing follow-up evaluations from his hematologist.

## Discussion

The cause of adrenal lymphoma is still poorly known. However, it is thought to have a multifactorial etiology, including immune-mediated infections, immune dysfunction that leads to deposits of hematologic tissue in the adrenal gland, and mutations in the p53 and c-kit genes [6]. For the diagnosis of DLBCL, the positivity of some molecular markers is necessary, including CD-10, Bcl-6, MUM1, MYC, and Bcl-2. It is also classified as a germinal or nongerminal center, the latter characterized by CD10-/BCL-6- or CD10-/MUM1+/ BCL6+ [2].

For our patient, examination of the surgical sample was the key to the definitive diagnosis of DLBCL, as it was double positive for MUM1 and Bcl-6 and negative for CD-10. Despite the enormous dimensions of the mass, the hormonal study excluded the presence of adrenal insufficiency, reinforcing the idea that there is no proven relationship between tumor size and organ dysfunction.

Regarding the prognosis, tumors larger than 8 cm, nongerminal center type, and Bcl-6 positive are associated with more rapidly progressive disease [7]. These lymphomas are usually bilateral (not unilateral as in this patient) and usually diagnosed in advanced stages. Chemotherapy was started immediately after diagnosis with the R-CHOP regimen. Rituximab-containing chemotherapy has shown promise by increasing the overall survival of patients with this disease [8]. It is necessary to emphasize that early diagnosis of this lymphoma, before adrenal insufficiency appears, contributes to decreasing the patient’s morbidity and mortality, just as it happened with our patient [9].

A series of extranodal lymphoma cases followed up between 2015 and 2017 revealed that all patients treated with R-CHOP achieved complete remission [10]. In most cases, surgery is waived, and chemotherapy alone is the most successful therapeutic regimen; however, in our case, with a nonfunctional tumor undiagnosed by noninvasive methods and with anatomical distortion, surgery was an essential step. We could not avoid nephrectomy due to the close relationship between the tumor, the kidney, and the adjacent vessels with an important hemorrhagic risk.

## Conclusions

This case describes a 68-year-old man diagnosed with a DLBCL of the adrenal gland who required excisional surgery for a definitive diagnosis after he presented to the Emergency Department with fatigue and anorexia. Given our patient’s indicators of a poor prognosis, this case emphasizes the importance of early diagnosis and rapid initiation of targeted treatment. This case also highlights the importance of R-CHOP therapy and his improved prognosis.

## References

[1] Vaidya A, Hamrahian A, Bancos I, Fleseriu M, Ghayee HK (2019). The evaluation of incidentally discovered adrenal masses. Endocr Pract.

[2] Wang Y, Ren Y, Ma L (2020). Clinical features of 50 patients with primary adrenal lymphoma. Front Endocrinol (Lausanne).

[3] Ollila TA, Olszewski AJ (2018). Extranodal diffuse large B cell lymphoma: molecular features, prognosis, and risk of central nervous system recurrence. Curr Treat Options Oncol.

[4] Pathmanathan K, Kodali V, Mohamad A (2020). Primary adrenal lymphoma: a case of hiccups. Oxf Med Case Reports.

[5] Kasaliwal R, Goroshi M, Khadilkar K (2015). Primary adrenal lymphoma: a single-center experience. Endocr Pract.

[6] Chen P, Jin L, Yang Y, Ni L, Yang S, Lai Y (2017). Bilateral primary adrenal diffuse large B cell lymphoma without adrenal insufficiency: a case report and review of the literature. Mol Clin Oncol.

[7] Li S, Wang Z, Wu Z, Zhuang H, Xu Y (2019). Clinical characteristics and outcomes of primary adrenal diffuse large B cell lymphoma in a large contemporary cohort: a SEER-based analysis. Ann Hematol.

[8] Ram N, Rashid O, Farooq S, Ulhaq I, Islam N (2017). Primary adrenal non-Hodgkin lymphoma: a case report and review of the literature. J Med Case Rep.

[9] Kacem K, Zriba S, Lakhal RB (2014). Primary adrenal lymphoma. Turk J Haematol.

[10] Suzuki Y, Sakakibara A, Shimada K (2019). Immune evasion-related extranodal large B-cell lymphoma: a report of six patients with neoplastic PD-L1-positive extranodal diffuse large B-cell lymphoma. Pathol Int.

